# Missing data on accessibility of children’s medicines

**DOI:** 10.2471/BLT.22.288137

**Published:** 2022-09-02

**Authors:** Iris R Joosse, Aukje K Mantel-Teeuwisse, Veronika J Wirtz, Fatima Suleman, Hendrika A van den Ham

**Affiliations:** a Utrecht World Health Organization Collaborating Centre for Pharmaceutical Policy and Regulation, Division of Pharmacoepidemiology and Clinical Pharmacology, Utrecht University, Universiteitsweg 99, 3584 CG, Utrecht, Netherlands.; b Department of Global Health, Boston University School of Public Health, Boston, United States of America.; c World Health Organization Collaborating Centre for Pharmaceutical Policy and Evidence Based Practice, University of KwaZulu-Natal, Durban, South Africa.

## Abstract

Child-appropriate medicines are essential for the safe and effective treatment of children, yet we have observed a large gap in the data required to adequately monitor access to these medicines. We have examined data on the availability and pricing of child-appropriate medicines across 50 surveys. Child-appropriate medicines for nine out of 12 priority diseases in children were infrequently surveyed or not at all. A similar data deficit on age-appropriate medicines is detectable in the broader scientific literature. We also note that existing instruments for collecting data on the availability or prices of medicines are limited in their ability to generate the required data for children. We have identified four priorities as key for improved monitoring of access to medicines for children: (i) dedicated child medicine surveys are needed on availability and prices of child-appropriate medicines; (ii) standardized survey instruments should include age-appropriate medicines and dosages; (iii) health facility service readiness survey tools should include the collection of data on the price of child-appropriate medicines in addition to the availability of medicines; and (iv) sustainable development goal indicator 3.b.3 should be modified to enable the monitoring of access to medicines for children. These deficiencies need to be addressed to ensure the monitoring of access to child medicines as part of the sustainable development goal agenda for 2030 and to implement appropriate interventions for improving access for this vulnerable population.

## Résumé

Disposer de médicaments adaptés aux enfants est essentiel à l'administration d'un traitement sûr et efficace. Pourtant, nous avons observé de vastes lacunes dans les données requises pour évaluer l'accès à ces médicaments. Nous avons passé 50 enquêtes au crible, à la recherche d'informations sur la disponibilité et le prix des médicaments pédiatriques. Dans le cas de neuf maladies infantiles prioritaires sur douze, les médicaments adaptés aux enfants n'étaient pas ou peu étudiés. Même constat dans le contexte plus large de la littérature scientifique. Nous avons également remarqué que les instruments servant à récolter des données sur la disponibilité ou le prix des médicaments avaient leurs limites et ne permettaient pas d'obtenir les informations requises concernant les enfants. Nous avons identifié quatre priorités majeures en vue d'améliorer la surveillance de l'accès aux médicaments pédiatriques: (i) la réalisation d'enquêtes sur les médicaments pédiatriques afin d'en connaître la disponibilité et le prix; (ii) l'intégration des médicaments et dosages adéquats dans les instruments d'enquête standard; (iii) outre la disponibilité, la prise en compte du prix des médicaments à usage pédiatrique dans les outils d'évaluation de l'état de préparation des services au sein des établissements de santé; et enfin, (iv) la modification de l'indicateur 3.b.3 des objectifs de développement durable, qui prévoirait dès lors un contrôle de l'accès aux médicaments adaptés aux enfants. Ces lacunes doivent être comblées pour assurer un suivi en matière d'accès aux médicaments pédiatriques dans le cadre du Programme de développement durable à l'horizon 2030, mais aussi pour adopter les mesures correspondantes afin d'améliorer la prise en charge de cette population vulnérable.

## Resumen

Los medicamentos indicados para los niños son esenciales para su tratamiento seguro y eficaz, pero se ha observado un gran vacío en los datos necesarios para supervisar de manera adecuada el acceso a estos medicamentos. Se han analizado los datos sobre la disponibilidad y el precio de los medicamentos indicados para los niños en 50 encuestas. Estos medicamentos para nueve de las 12 enfermedades prioritarias infantiles se encuestaron con poca frecuencia o no se encuestaron en absoluto. En la literatura científica más general, se detecta un déficit de datos similar sobre los medicamentos adecuados para la edad. También se observa que los instrumentos existentes para recopilar los datos sobre la disponibilidad o los precios de los medicamentos son limitados en su capacidad para generar los datos necesarios en el caso de los niños. Se han identificado cuatro prioridades para mejorar el seguimiento del acceso a los medicamentos pediátricos: (i) se necesitan encuestas específicas sobre la disponibilidad y los precios de los medicamentos indicados para los niños; (ii) los instrumentos de encuesta estandarizados deben incluir medicamentos y dosis adecuados para la edad; (iii) las herramientas de encuesta sobre la disponibilidad de los servicios sanitarios deben incluir la recopilación de los datos sobre el precio de los medicamentos indicados para los niños, además de la disponibilidad de los medicamentos; y (iv) el indicador 3.b.3 del Objetivo de Desarrollo Sostenible se debe modificar para permitir el seguimiento del acceso a los medicamentos pediátricos. Es preciso solucionar estas deficiencias para garantizar el seguimiento del acceso a los medicamentos pediátricos como parte de la agenda de los objetivos de desarrollo sostenible para 2030 y aplicar las intervenciones adecuadas para mejorar el acceso de esta población vulnerable.

## ملخص

تعد الأدوية المناسبة للأطفال ضرورية للعلاج الآمن والفعال للأطفال، ومع ذلك فقد لاحظنا وجود فجوة واسعة في البيانات المطلوبة لمراقبة الحصول على هذه الأدوية بشكل مناسب. لقد قمنا بفحص البيانات المتعلقة بتوافر وتسعير الأدوية المناسبة للأطفال عن طريق 50 استطلاعًا للرأي. لم يتم إجراء استطلاع للرأي بخصوص الأدوية المناسبة للأطفال لعلاج 9 من أصل 12 من الأمراض ذات الأولوية للأطفال بشكل متواتر، أو لم يتم إجراء استطلاع للرأي عليها على الإطلاق. يمكن اكتشاف عجز مماثل في البيانات المتعلقة بالأدوية المناسبة للعمر في المنشورات العلمية الأوسع نطاقًا. كما نلاحظ أيضًا أن الأدوات الحالية لتجميع البيانات حول توافر الأدوية أو أسعارها محدودة من حيث قدرتها على توليد البيانات المطلوبة للأطفال. لقد حددنا أربع أولويات باعتبارها أساسية لتحسين مراقبة حصول الأطفال على الأدوية: (1) الحاجة إلى استطلاعات رأي مخصصة لطب الأطفال بخصوص توافر الأدوية المناسبة للأطفال وأسعارها؛ و(2) يجب أن تشمل أدوات استطلاع الرأي القياسية الأدوية والجرعات المناسبة للعمر؛ و(3) إن أدوات استطلاع مدى جاهزية خدمة المرافق الصحية يجب أن تتضمن تجميع البيانات عن أسعار الأدوية المناسبة للأطفال بالإضافة إلى توافر الأدوية؛ و(4) يجب تعديل مؤشر هدف التنمية المستدامة 3.b.3 لتمكين مراقبة حصول الأطفال على الأدوية. يجب معالجة أوجه القصور هذه لضمان مراقبة الحصول على أدوية الأطفال كجزء من أجندة أهداف التنمية المستدامة لعام 2030، ولتنفيذ التدخلات المناسبة لتحسين الحصول لدى هذه الفئة السكانية المهمشة.

## 摘要

儿童适用药对于儿童的安全和有效治疗至关重要，然而我们发现用于适当监测这些药品可获取性所必需的数据相当缺乏。我们基于 50 项调查检查了有关儿童适用药可获取性和价格的数据。在 12 种重大儿童疾病中，用于治疗其中 9 种疾病的儿童适用药很少或根本未被纳入调查范围。在查阅更多科学文献的过程中，我们也发现了类似的适龄药物数据不足现象。我们还注意到，用于收集药品可获取性或价格相关数据的现有工具无法有效生成所需的儿童相关数据。我们确定了加强监测儿童药品可获取性的四个关键优先事项：(i) 需要基于儿童适用药可获取性和价格专门开展儿童药品调查；(ii) 标准化的调查工具应具有调查适龄药物和剂量的功能；(iii) 卫生设施服务准备情况调查工具应具有收集有关儿童适用药价格以及药物可获取性的数据的功能；以及 (iv) 应修改可持续发展目标指标 3.b.3，以确保能够监测儿童适用药的获取情况。需要解决这些问题才能确保将监测儿童药品可获取性纳入 2030 年可持续发展目标议题，并采取适当的干预措施改善这一弱势群体获取药物的情况。

## Резюме

Лекарственные средства, подходящие для детей, имеют важное значение для безопасного и эффективного лечения этой категории пациентов, однако наблюдается большой пробел в данных, необходимых для надлежащего мониторинга доступа к таким лекарственным средствам. Авторы изучили данные 50 исследований о ценах на лекарственные средства, подходящие для детей, а также о наличии таких лекарственных средств. Исследования таких средств для лечения девяти из 12 приоритетных заболеваний у детей проводились крайне редко или совсем не проводились. Аналогичный дефицит данных о соответствующих возрасту лекарственных средствах можно обнаружить в более широкой научной литературе. Авторы также отмечают, что существующие инструменты для сбора данных о ценах на лекарственные средства, а также об их наличии ограничены в своих возможностях получить необходимые данные о детях. Авторы определили четыре основных приоритета для улучшения мониторинга доступа к лекарственным средствам для детей: (i) необходимы специальные исследования лекарственных средств для детей, касающиеся наличия и цен на такие средства; (ii) в стандартизированные средства исследования нужно включить лекарственные средства и дозировки, соответствующие возрасту; (iii) средства исследования готовности медицинских учреждений к предоставлению услуг должны включать сбор данных о ценах на лекарственные средства для детей, а также об их наличии; (iv) показатель цели в области устойчивого развития 3.b.3 следует изменить, чтобы обеспечить мониторинг доступа к лекарственным средствам для детей. Эти недостатки необходимо устранить, чтобы обеспечить мониторинг доступа к лекарственным средствам для детей в рамках повестки дня по достижению целей в области устойчивого развития до 2030 г. и принять соответствующие меры для улучшения доступа для этой уязвимой группы населения.

## Introduction

**Fig. 1:**
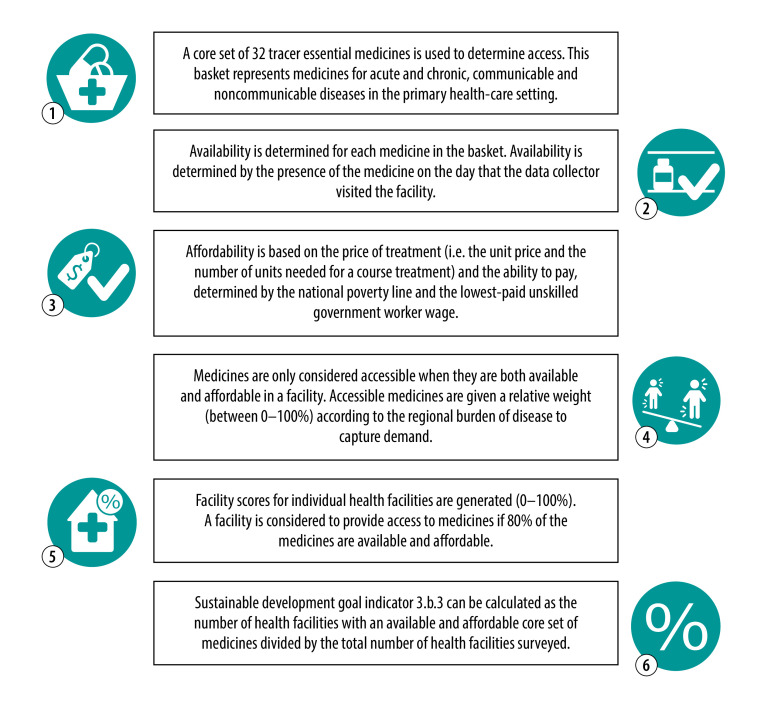
How sustainable development goal indicator 3.b.3 is used for measuring access to medicines

Note: Adapted from United Nations, 2019.^2^

The importance of access to available and affordable essential medicines for all is embodied in targets 3.8 and 3.b of the sustainable development goals (SDGs) and remains a priority on the international agenda.^1^ Data on the availability and prices of medicines are considered pivotal for measuring progress on these SDGs. The key indicator to assess country progress on target 3.b (indicator 3.b.3) requires both availability and price as inputs (Fig. 1).^2^ The outcomes of the indicators are meant to guide national and international efforts to improve people’s access to medicines.^3^

However, research efforts have traditionally focused on measuring access to medicines for the general population, without particular consideration for medicines for children. As a result, we note that there is a major gap in our understanding of accessibility of medicines for children. The gap is manifested in two ways: first, validated surveys dedicated to medicines for children is lacking; second, surveys whose results are made available in the public domain have not sufficiently covered child-appropriate medicines. In addition to this data gap, the main indicator to measure access to medicines (SDG indicator 3.b.3) is primarily aimed at adults. These deficiencies impede the monitoring and understanding of accessibility of paediatric medicines and thereby the possibility for policy-makers to implement appropriate interventions. We discuss here the extent of the data gaps and propose ways to address the gaps.

## Age-appropriate medicines

Children require medicines that are age-appropriate. Differences in the pharmacokinetic and pharmacodynamic profile of children and adults mean that children require different dosage strengths. There is also a need for preparations that are easy to administer, contain excipients that are safe for children, are better accepted by children, and enable flexible dosing.^4^ In recent years there has been a shift away from liquid formulations to solid oral dosage forms.^5^ However, most traditional solid oral preparations are unsuitable for children younger than 6 years due to the risk of choking and difficulties with swallowing.^6^ Additionally, manipulation of existing dosage forms (such as breaking, crushing or diluting) may cause harmful dosing errors.^7^ Child-appropriate medicines – such as orodispersible or chewable tablets and possibly oral liquids or rectal formulations – are thus required to achieve effective and safe treatment and often cannot be replaced by medicines for adults. Accordingly, it is essential that child-appropriate medicines are monitored for their availability and affordability.

## Data deficit

In our attempts to assess the accessibility of medicines for children we observed that there is a large deficiency in data on the availability and price of child-appropriate medicines. To illustrate the extensiveness of the data gap on child medicines, we screened surveys using the standardized World Health Organization (WHO)-Health Action International method on the availability and prices of child-appropriate medicines used in treating diseases with the highest burden in children. These survey methods are regarded as a gold standard when studying the availability and price of medicines, and the survey results have been widely used to assess progress towards the millennium development goals.^8^^,^^9^

We screened surveys on child-appropriate medicines for 12 priority diseases in children. We selected 10 diseases that are associated with the highest absolute burden of disease expressed in disability-adjusted life years for children aged 0–14 years according to the Global Health Estimates^10^ and treatable with medicines from the WHO essential medicines list for children.^11^ We also selected two other diseases ‒ pain and palliative care, and vitamin K-associated bleeding ‒ which are not included in the Global Health Estimates, but nevertheless represent priority diseases. We defined appropriate medicines as first-choice medicines in primary care according to the uses described in the essential medicines list for children.^11^ Dosage forms that we considered child-appropriate included inhalers, oral liquids, injections, powders for dissolving, suppositories and chewable or dispersible tablets. If none of the dosage forms above were listed in the essential medicines list for children, we also considered tablets or capsules as appropriate. We screened surveys that were conducted according to the WHO-Health Action International method for child-appropriate medicines and were published on the initiative’s website.^12^ We started screening at the most recent surveys.

In total, we screened 50 surveys, conducted between 2001 and 2015 and across 43 countries in all six WHO regions (Box 1). A single survey was specifically dedicated to child medicines, whereas no specific age group was targeted in the other 49 surveys. Table 1 shows the number and percentage of surveys that included at least one child-appropriate medicine for each of the 12 priority diseases in children. Our findings show that child-appropriate medicines for nine out of 12 diseases were only sporadically surveyed or not at all, including medicines for treatment of tuberculosis and iron deficiency anaemia. The better results for asthma medication and antibiotics are probably because the formulations of these medicines (such as inhalers and injections) are used by adults as well. Noteworthy, however, (child) spacers for inhaled medications were not part of our screening. Additionally, these results may not be applied to individual medicines, as the results in Table 1 are grouped by indication. To illustrate, ampicillin was included in only eight of 50 surveys, and even a key antibiotic such as amoxicillin (or amoxicillin plus clavulanic acid) was surveyed as a child-appropriate formulation in just half of the surveys (26 of 50).

Box 1 **World Health Organization-Health Action International surveys screened for child-appropriate medicines for treatment of common childhood diseases, by country and year**

**African Region**

Burkina Faso, 2009; Burundi, 2013; Ethiopia, 2013; Uganda, 2015; United Republic of Tanzania, 2012.

**Region of the Americas**

Bolivia (Plurinational State of), 2008; Brazil, 2008; Colombia, 2008; Haiti, 2011; Mexico, 2009; United States of America, 2015.

**Eastern Mediterranean Region**

Afghanistan, 2011; Egypt, 2013; Iran (Islamic Republic of), 2007; Jordan, 2004; Kuwait, 2004; Lebanon, 2004; Lebanon, 2013; Morocco, 2004; Oman, 2007; Pakistan, 2004; Saudi Arabia, 2015; Sudan, 2012; Sudan, 2013; Syrian Arab Republic, 2003; Tunisia, 2004; United Arab Emirates, 2006; Yemen, 2006.

**European Region**

Armenia, 2001; Kazakhstan, 2004; Kyrgyzstan, 2005; Kyrgyzstan, 2010; Kyrgyzstan, 2015; Republic of Moldova, 2011; Russian Federation, 2011; Tajikistan, 2005; Tajikistan, 2013; Ukraine, 2007; Ukraine, 2012; Uzbekistan, 2004.

**South-East Asia Region**

India, 2005; India, 2011; Indonesia, 2010; Sri Lanka, 2001; Thailand, 2006.

**Western Pacific Region**

China, 2012;^a^ Lao People's Democratic Republic, 2013; Malaysia, 2004; Mongolia, 2012; Philippines, 2008.

^a^ Survey dedicated to paediatric medicines.

**Table 1:** World Health Organization-Health Action International surveys that covered child-appropriate medicines for treatment of common childhood diseases

Disease	No. (%) of surveys of child-appropriate medicines (*n* = 50)	Associated disease burden (ranked)^a^	Age group most affected
Asthma	49 (98)	8	5–14 years
Bacterial infectious diseases^b^	45 (90)	1	All ages
Pain and palliative care	24 (48)	–^c^	All ages
Diarrhoeal diseases	13 (26)	2	All ages
Malaria	7 (14)	3	1 month–14 years
Epilepsy	7 (14)	9	1 month–14 years
Measles	3 (6)	5	1 month–5 years
Migraine	1 (2)	10	5–14 years
Tuberculosis	0 (0)	4	1 month–14 years
Iron deficiency anaemia	0 (0)	6	1 month–14 years
HIV/AIDS	0 (0)	7	All ages
Vitamin K-deficiency bleeding	0 (0)	–^c^	Neonates

AIDS: acquired immunodeficiency syndrome; HIV: human immunodeficiency virus infection; WHO: World Health Organization.

^a^ We selected diseases with the highest burden of disease in children (in disability-adjusted life years) from the Global Health Estimates.^10^

^b^ Bacterial infectious diseases is an aggregated term for several prevalent infectious diseases with bacterial origin (such as lower respiratory infections, neonatal sepsis, meningitis, pertussis and syphilis).

^c^ Dashes indicate that these diseases are not associated with a burden in the Global Health Estimates, so no rank can be assigned.

Note: Dosage forms considered child-appropriate were: inhalers, injections, oral liquids, powders for dissolving, suppositories, and chewable or (oro)dispersible tablets. If none of the dosage forms above were listed in the WHO essential medicines list for children,^11^ tablets or capsules were also considered appropriate.

A similar data deficit and lack of attention to age-appropriate formulations is detectable in the broader scientific literature. A recent systematic review on accessibility of child medicines identified only 18 surveys that included data on the availability, price or affordability of paediatric medicines, out of 4732 records screened.^13^ There were only 11 studies that reported both the availability and price of medicines. Of note, one of these surveys was also recorded in the Health Action International database and included in our own sample of surveys screened (China, 2012). The surveys identified in the systematic review were conducted from 2009 to 2019 and included studies from eight different countries.^13^ We judged that seven of 18 studies included surveys of limited significance for measuring accessibility of medicines in a country, as they (i) focused on one disease area only (such as cardiovascular medicine or cancer medicine), (ii) were studies of formulations that were often not age-appropriate (such as traditional solid oral dosage forms versus oral liquids or flexible oral dosage forms), or (iii) solely looked at active ingredients and not formulations.

## Limitations of tools

Despite being considered the gold standard, the WHO-Health Action International survey type has been less used since 2015. WHO has instead been looking at other means to collect data on the availability and pricing of medicines, partly to promote leaner data collection and analysis methods. The WHO Essential Medicines and Health Products Price and Availability Monitoring mobile application (MedMon) is such an instrument.^14^ This tool was developed for rapid and flexible data collection and analysis, and should facilitate more routine monitoring. Nonetheless, widespread implementation of this promising tool has been delayed, despite several successful pilot studies.

A WHO-recommended instrument for assessing health facility performance is the Service Availability and Readiness Assessment survey, a tool designed through collaboration between WHO and the United States Agency for International Development (USAID).^15^ Although essential medicines are only a small part of this tool’s scope, it has been suggested that these surveys could nevertheless be an important data source for monitoring access to medicines. However, the relevance of data from these surveys for children is very limited because this survey type does not specify which formulations should be surveyed, or which formulations are age-appropriate. More importantly, collection of price data is not part of this tool. With affordability being a core component of accessibility to medicines, the applicability of this tool in monitoring accessibility of child medicines is limited.

Another tool for collecting data on essential medicines, and a predecessor of the Service Availability and Readiness Assessment, is the Service Provision Assessment within the Demographic and Health Surveys programme, funded by USAID and other partners.^16^ Service Provision Assessment was designed to gather data on a range of health facility services and their quality, with child and maternal health being one of the key topics assessed in these surveys. Although the dosage form of medicines for surveys is specified, the number of medicines that are relevant for children is sparse. Similar to Service Availability and Readiness Assessment surveys, Service Provision Assessment does not include collection of data on medicine prices.

Apart from original data collection, secondary data to benchmark affordability are also lacking. A widely used standard reference to benchmark medicines prices, the Management Sciences for Health International Medical Products Price Guide, has not been updated since 2015.^17^ In addition, commercial data sets such as those provided by IQVIA® (IQVIA Inc., Durham, United States of America) may include meaningful data in terms of medicines’ sales and utilization, but these data sets are generally not publicly available, although some countries may have purchased a license for access. An overview of data collection tools and sources can be found in Table 2.

**Table 2:** Characteristics and limitations of data collection tools

Tool	Main characteristics	Limitations
**Primary data collection tools **		
Standardized WHO-Health Action International surveys^8^	Designed to collect and analyse data on availability and prices of medicines	Paper-based tool Little used since 2015
WHO Essential Medicines and Health Products Price and Availability Monitoring mobile application (MedMon)^14^	Electronic tool designed to collect and analyse data on availability and prices of medicines	Tool currently unavailable to the public due to modifications being implemented
Service Availability and Readiness Assessment surveys^15^	Designed to collect data on availability of medicines at facility level, among other facility services	Data on medicine prices not collected No data on private sector outlets Tool does not collect data on age-appropriate medicines
Service Provision Assessment surveys^16^	Designed to collect data on availability of medicines at facility level, among other facility services	Data on medicine prices not collected No data on private sector outlets Number of age-appropriate medicines limited
**Secondary data sources**		
International Medical Products Price Guide^17^	Includes international comparative price data on medicines	Not updated since 2015
Electronic medical records, sales data, claims data and hospital data (such as IQVIA® data sets)^a^	Real-world data routinely collected from a variety of sources on sales and use of medicines, and other health data	Available on purchase Data on availability not recorded

WHO: World Health Organization.

^a^ IQVIA Inc., Durham, United States of America.

## Closing the gap

In an effort to close the gap in accessibility between adult and child medicines, a landmark resolution called *Better medicines for children* was adopted by the World Health Assembly in 2007.^18^ This resolution identified several areas that needed to be addressed to close the gap and requested WHO to intensify their efforts in making safe and effective medicines as widely available for children as for adults. Since this resolution, WHO has invested in comprehensive activities to improve access for children, including establishing the first WHO model list of essential medicines for children in 2007.^11^^,^^19^ This initiative was 30 years after the first essential medicines list, which included some medicines for children but failed to systematically consider medicines for this vulnerable population at the time. Other milestones since the resolution included the Make Medicines Child Size campaign (2007), the development of a model formulary for children (2010), the establishing of a priority list of essential medicines for women and children (2011), and updated treatment guidelines (2013).^20^^–^^23^

Despite the increased attention on child-appropriate medicines globally, data collection on the subject still lags behind that for adult medicines. In fact, the SDG indicator that was designed in 2017 to measure access to medicines fails to address the needs of children.^2^ This current lack of a method and an indicator that combine availability and affordability of medicines for children in a single measure is an important gap that needs to be filled. For the indicator to be appropriate for measuring access to medicines for children, the method should include a basket of medicines with a broader selection of medicines that are relevant to children, including age-appropriate dosage forms and strengths (Fig. 1, step 1). Additionally, a novel measure should be developed for the number of units that are needed for a course of treatment for children (Fig. 1, step 3), to substitute the defined daily dosage that is currently used in the calculations but is applicable to adults only. Without such adjustment, measurement for children would not be possible. Furthermore, for this prospective child indicator to be of real value, the corresponding data on availability and prices of child medicines are required. This necessity is highlighted in the tier classification for global SDG indicators, which requires that – for a so-called tier 1 indicator – both a method is established, and the data are produced regularly by countries.^24^

We have identified the following four priorities for adequate monitoring of access to medicines for children. First, we call for urgent action to fill the current data gap, as countries have to report each year on their progress towards the 2030 SDGs. Surveys that are conducted for the general population should stratify results by child and adult medicines. Second, standardized survey types for collecting data on the availability and prices of medicines – such as the surveys using the WHO-Health Action International method – should provide guidance and the tools for collecting the required data on child-appropriate medicines. These survey instruments should include a broad range of priority medicines for children of different ages, alongside those for adults. Special attention should be paid to the inclusion of flexible oral solid-dosage forms and other child-appropriate dosage forms. New technologies such as the WHO MedMon application may provide opportunities for gathering the appropriate data and should be implemented without further delay.^14^ Third, any routine assessment of facility readiness such as Service Availability and Readiness Assessment and Service Provision Assessment surveys should include assessment of the affordability of essential medicines for adults and children as well as their availability.^15^^,^^16^ Fourth, the SDG 3.b.3 indicator for measuring access to medicines as a combination of availability and affordability needs to be adjusted to make it appropriate for child medication.^2^

## Implications

Because of the unique requirements of children, data on adult medicines do not provide an insight into access to medicines for children. We believe that swift action is needed to include child medicines in national surveys. If this is not done soon, an important window of opportunity will be missed to improve accountability and transparency in progress towards access to medicines – for both adults and children – as part of the 2030 SDGs agenda. If medicines were to be dropped from the overall progress report on SDGs, it would be the second time that the global public health community has failed to report on access to medicines.^25^

We observed that child-appropriate medicines are neglected when measuring accessibility to medicines. Although the data deficit we discuss above may not provide a complete overview of the available data on children’s medicines, it nonetheless highlights the gaps in these types of data. This situation is concerning; without sufficient and appropriate data to inform us, we cannot identify potential barriers to access to medicines and to accomplish real change. Children have no voice to advocate for themselves. Who will advocate on their behalf for adequate data to improve and trace access to child-appropriate medicines?
